# Supplementary material for: A visualization reporter system for characterizing antibiotic biosynthetic gene clusters expression with high-sensitivity
Source: Commun Biol. 2022 Sep 2;5:901. doi: 10.1038/s42003-022-03832-9 (PMC9440138; doi:10.1038/s42003-022-03832-9)
Supplement: Supplementary file 2 — Supplementary Information [file 42003_2022_3832_MOESM2_ESM.pdf]

Supplementary Information

A visualization reporter system for characterizing antibiotic biosynthetic gene clusters expression with high-sensitivity

Xiang Liu[1,2,#], Jine Li[1,#], Yue Li[1], Junyue Li[1,2], Huiying Sun[1,2], Jiazhen Zheng[1,2], Jihui Zhang[1]* & Huarong Tan[1,2]*

[1]State Key Laboratory of Microbial Resources, Institute of Microbiology, Chinese Academy of Sciences, Beijing 100101, China

[2]College of Life Sciences, University of Chinese Academy of Sciences, Beijing 100049, China

[#]These authors contributed equally to this work.

*Corresponding authors, e-mail: zhang.jihui@im.ac.cn (Jihui Zhang); tanhr@im.ac.cn (Huarong Tan)

Supplementary figures

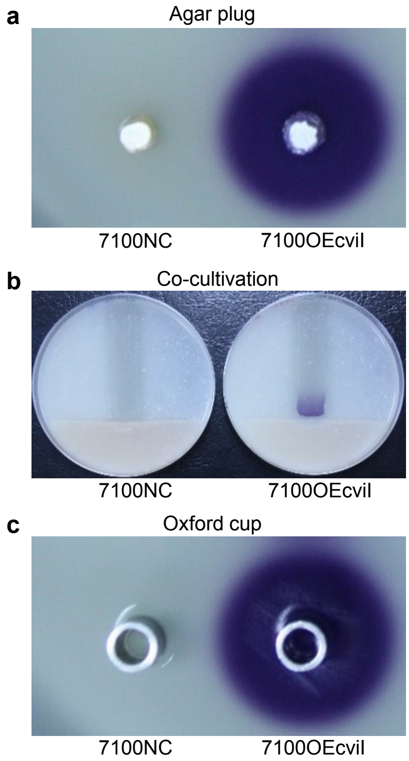

Supplementary Fig. 1. Different detection methods of AHL production in VRS-bAHL. **a** Agar plug method; **b** Co-cultivation method; **c** Oxford cup method. 7100OEcviI (*S. ansochromogenes* 7100 derivative strain with *cviI* overexpression driven by P_{hrdB} on pIJ10500K) was used to evaluate the effectiveness of these methods in comparison with 7100NC (*S. ansochromogenes* 7100 derivative strain harboring the blank plasmid pIJ10500K) as the negative control, and solid (**a**, **b**) or liquid MS medium (**c**) was used for cultivation. CV026 was used as the indicator strain. Data shown are representative of two independent experiments that displayed similar results.

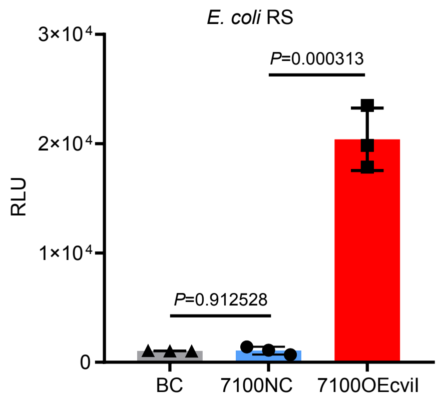

Supplementary Fig. 2. Detection of AHL using *E. coli* reporter strain. The *E. coli* reporter strain EJ532-4 constructed previously[1] was used to detect C6-HSL of 7100OEcviI in comparison with 7100NC as the negative control according to the intensity of bioluminescence induced by C6-HSL in 96-well plates. BC, blank control, EJ532-4 in LB medium. Individual data points are provided and the data are presented as means ± s.d. from three biologically independent samples. *P* value was calculated with two-tailed unpaired *t*-test. Source data are provided in Supplementary Data 10. All data shown are representative of two independent experiments that displayed similar results.

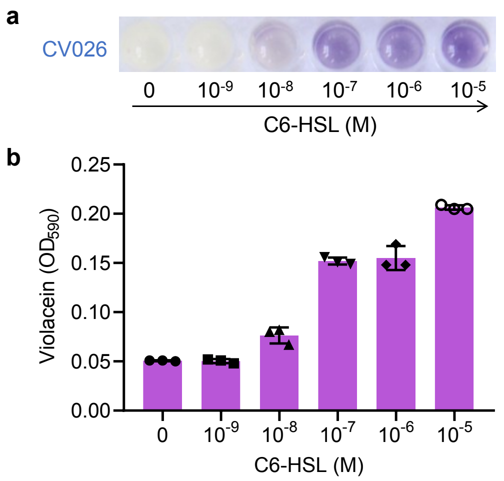

Supplementary Fig. 3. Dose-response curve of violacein production in CV026 induced by C6-HSL. **a** Violacein production in CV026 grown on LB soft agar medium in the presence of different concentrations of C6-HSL. Representative image of three biologically independent samples was displayed. **b** Quantitation of violacein production by measuring the optical density at 590 nm of the DMSO extract of violacein in each well. Individual data points are provided and the data are presented as means ± s.d. from three biologically independent samples. Source data of **b** are provided in Supplementary Data 11. In **a** and **b**, 96-well plates were used as previously described[1], and the data shown are representative of two independent experiments that displayed similar results.

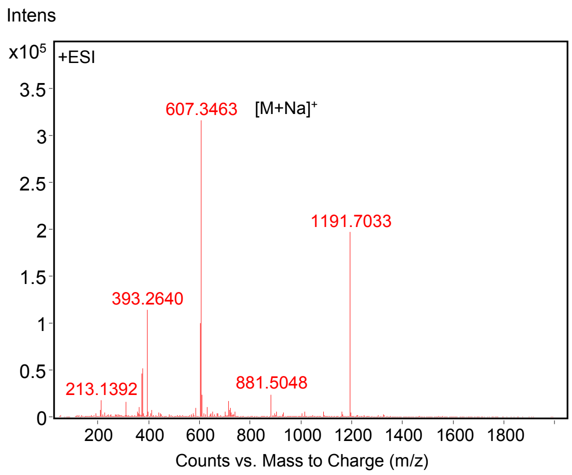

Supplementary Fig. 4. Mass spectrum of compound 2 in the extract of *S. ansochromogenes* 7100 grown on TSB medium.

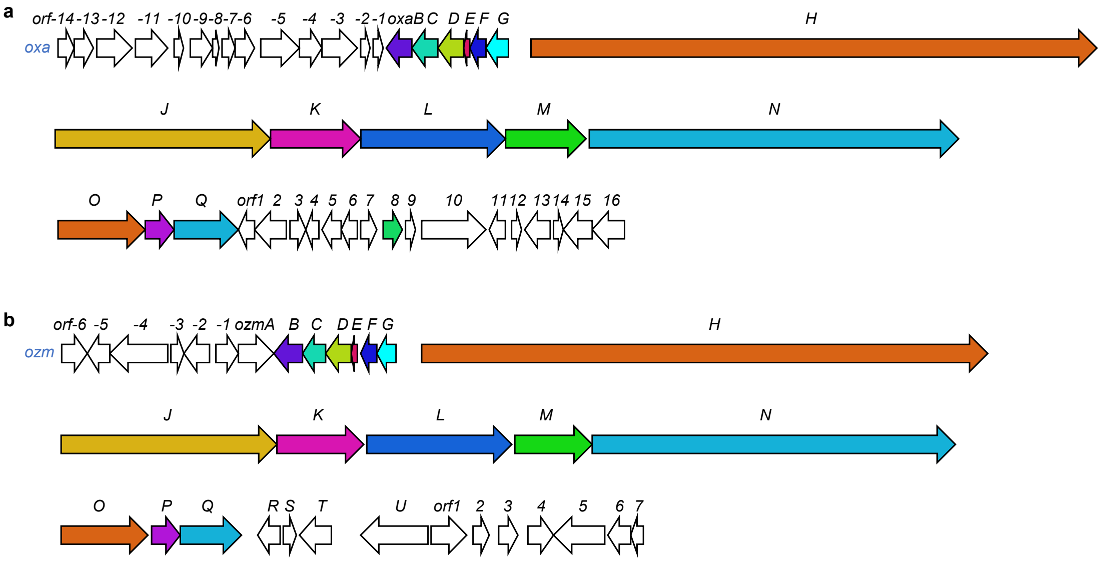

Supplementary Fig. 5. Organization and comparison of two oxazolomycin BGCs. **a** Organization of *oxa*, oxazolomycin BGC in *S. longshengensis* CGMCC 4.1101. **b** Organization of *ozm*, oxazolomycin BGC in *S. albus* JA3453[2]. The homologous genes in the two clusters are indicated with same color. Empty arrows indicate the genes whose corresponding homologs were not found in the other oxazolomycin BGC. In addition, each arrow represents one gene and its direction.

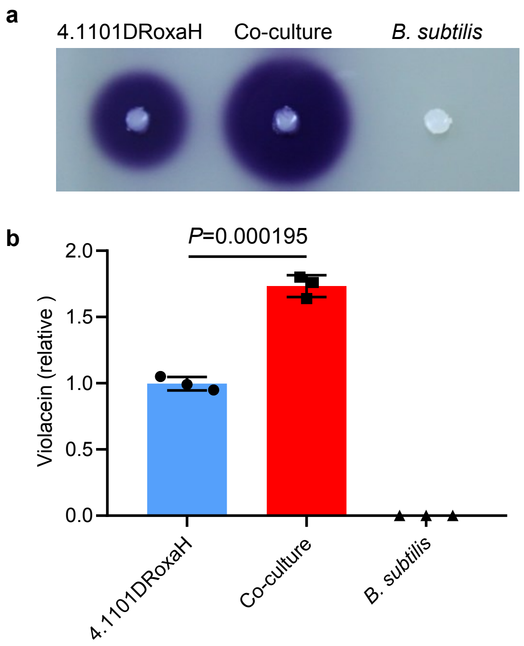

Supplementary Fig. 6. VRS-bAHL-guided characterization of *oxaH* transcription enhancement in *S. longshengensis* co-cultivated with *B. subtilis*. **a** Co-cultivation of 4.1101DRoxaH with *B. subtilis* and the detection of AHL production with CV026. Representative image of three biologically independent samples was displayed. **b** Quantitation of violacein production by the relative area of violacein zone with ImageJ software. All values were normalized to that of 4.1101DRoxaH. Individual data points are provided and the data are presented as means ± s.d. from three biologically independent samples. *P* value was calculated with two-tailed unpaired *t*-test. Source data of **b** are provided in Supplementary Data 12. Data shown above are representative of two independent experiments that displayed similar results.

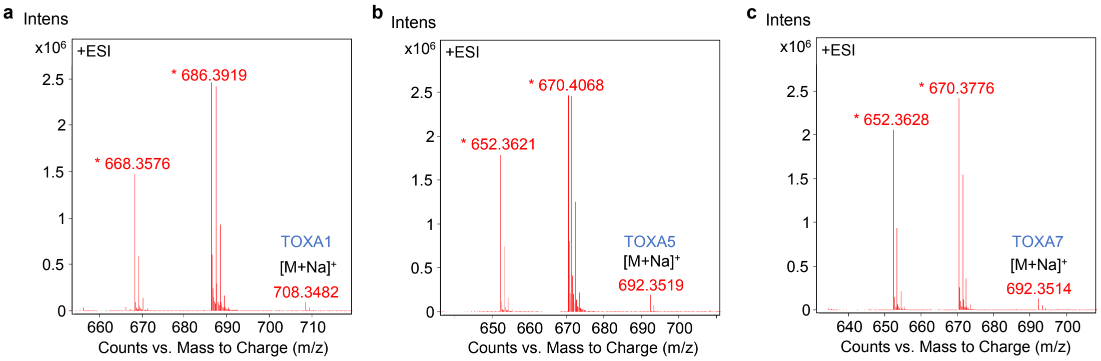

Supplementary Fig. 7. Mass spectra of TOXA1 (**a**), TOXA5 (**b**) and TOXA7 (**c**).

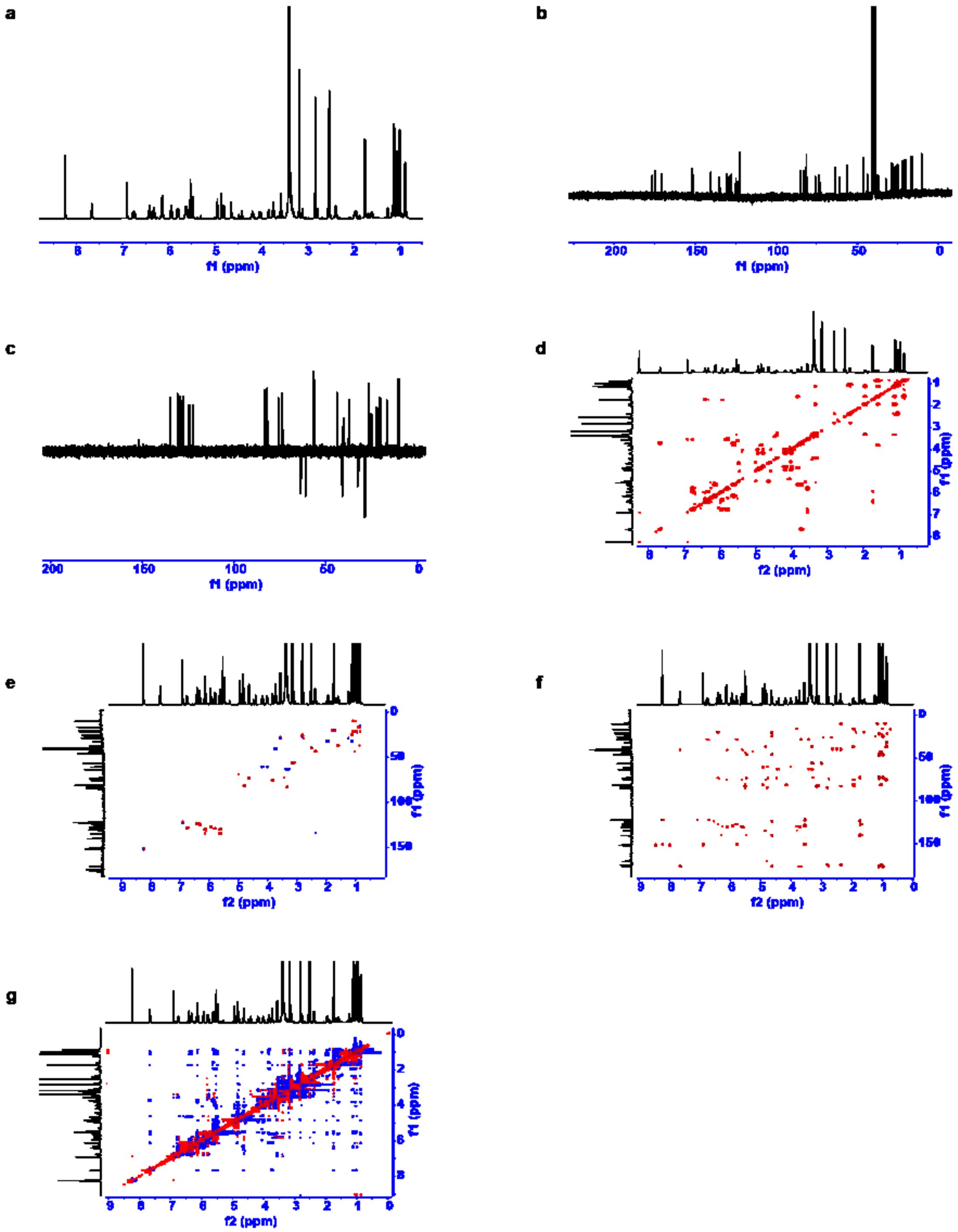

Supplementary Fig. 8. NMR spectra of TOXA1. **a** ^{1}H-NMR spectrum. **b** ^{13}C-NMR spectrum. **c** DEPT135 spectrum. **d** ^{1}H-^{1}H COSY spectrum. **e** ^{1}H-^{13}C HSQC spectrum. **f** ^{1}H-^{13}C HMBC spectrum. **g** ^{1}H-^{1}H ROESY spectrum.

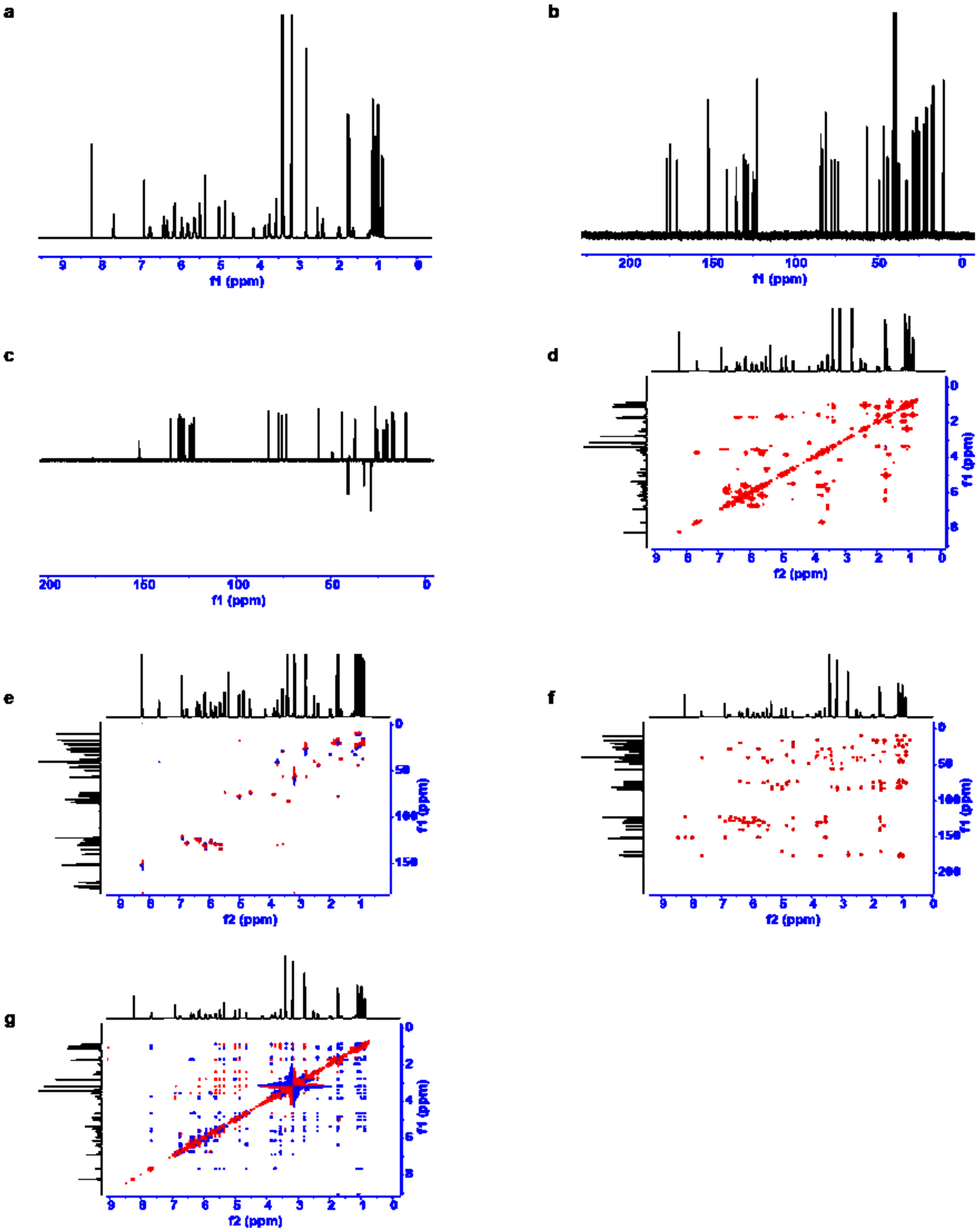

Supplementary Fig. 9. NMR spectra of TOXA5. **a** ^{1}H-NMR spectrum. **b** ^{13}C-NMR spectrum. **c** DEPT135 spectrum. **d** ^{1}H-^{1}H COSY spectrum. **e** ^{1}H-^{13}C HSQC spectrum. **f** ^{1}H-^{13}C HMBC spectrum. **g** ^{1}H-^{1}H ROESY spectrum.

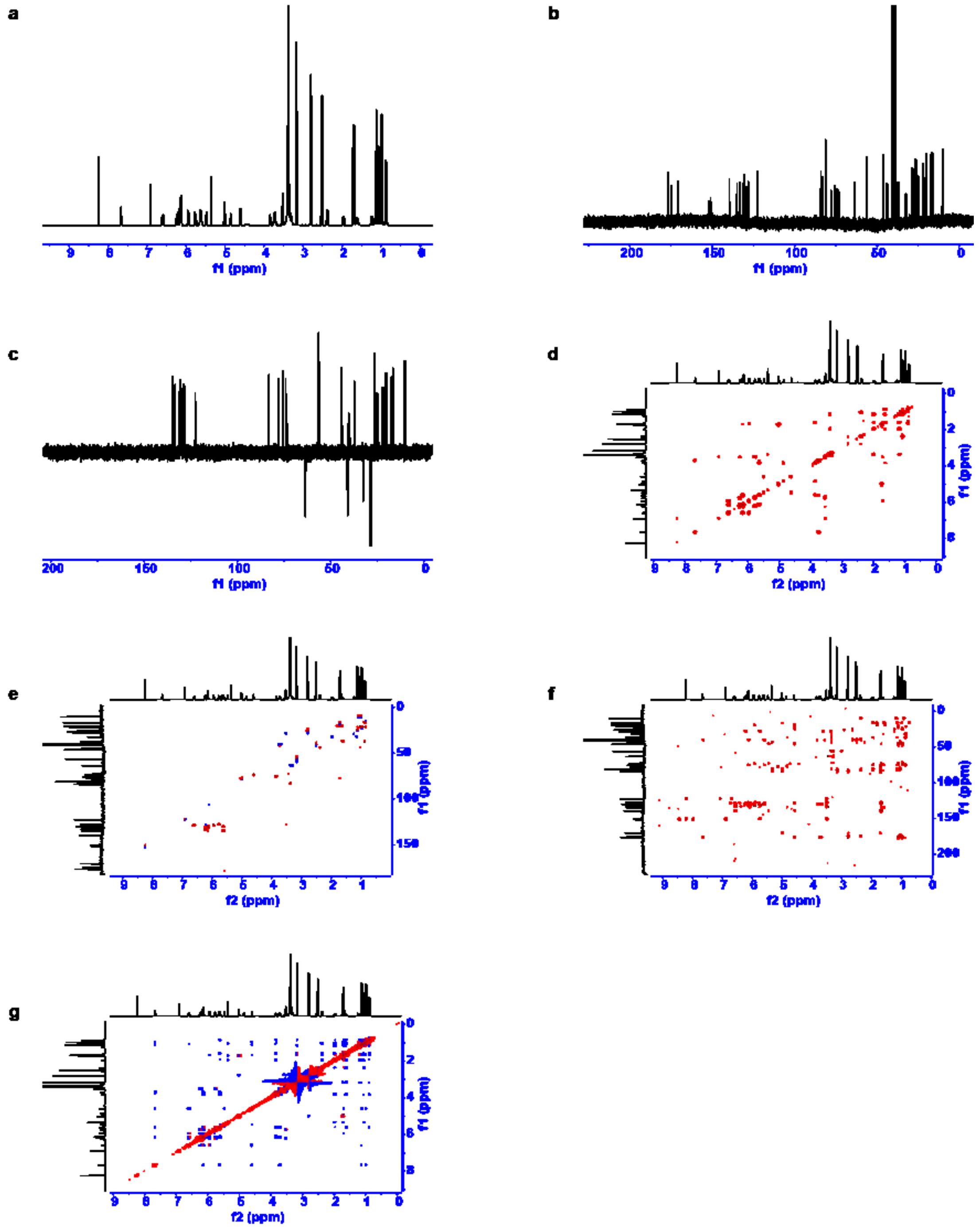

Supplementary Fig. 10. NMR spectra of TOXA7. **a** ^{1}H-NMR spectrum. **b** ^{13}C-NMR spectrum. **c** DEPT135 spectrum. **d** ^{1}H-^{1}H COSY spectrum. **e** ^{1}H-^{13}C HSQC spectrum. **f** ^{1}H-^{13}C HMBC spectrum. **g** ^{1}H-^{1}H ROESY spectrum.

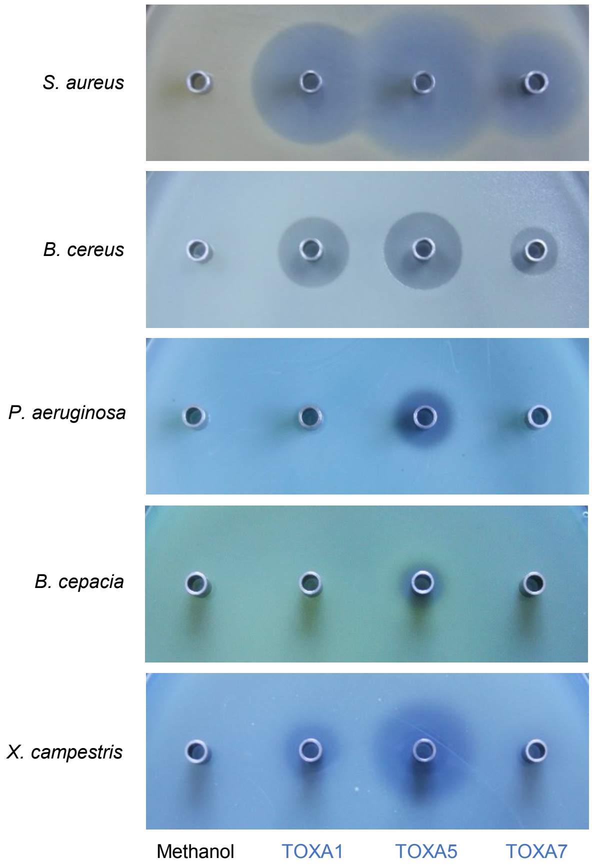

Supplementary Fig. 11. Antibacterial assays of TOXA1, TOXA5 and TOXA7. 40 μg of each compound dissolved in 50 μL of methanol was used for antibacterial assays against *Staphylococcus aureus*, *Bacillus cereus*, *Pseudomonas aeruginosa*, *Burkholderia cepacia* and *Xanthomonas campestris*, respectively. 50 μL of methanol was used as blank control. Data shown above are representative of two independent experiments that displayed similar results.

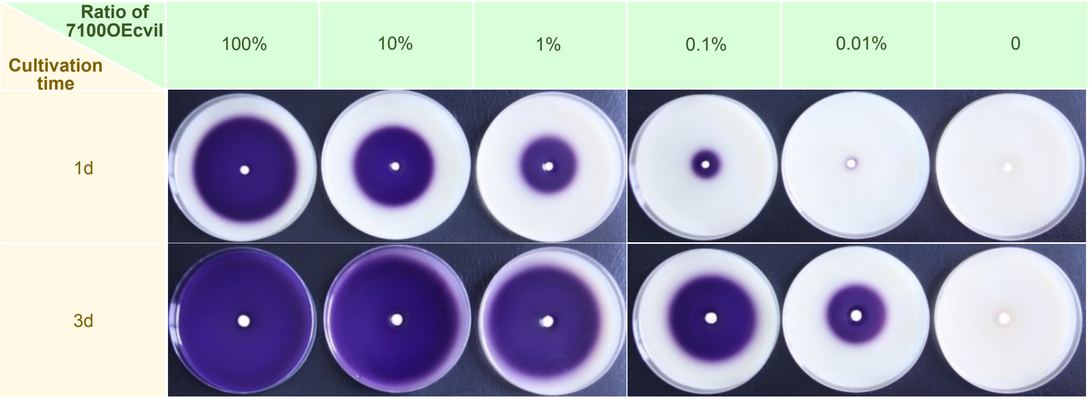

Supplementary Fig. 12. Evaluation of VRS-bAHL in pool testing. Spores of 7100OEcviI (derivative strain of *S. ansochromogenes* 7100 containing *cviI* overexpression plasmid driven by P_{hrdB} on pIJ10500K as positive control strain) were mixed with negative control strain 7100NC (*S. ansochromogenes* 7100 containing blank plasmid pIJ10500K) at different ratios and cultivated on MS agar medium for 1 day and 3 days, followed by AHL detection with CV026 through double-layer plate method. Data shown above are representative of two independent experiments that displayed similar results.

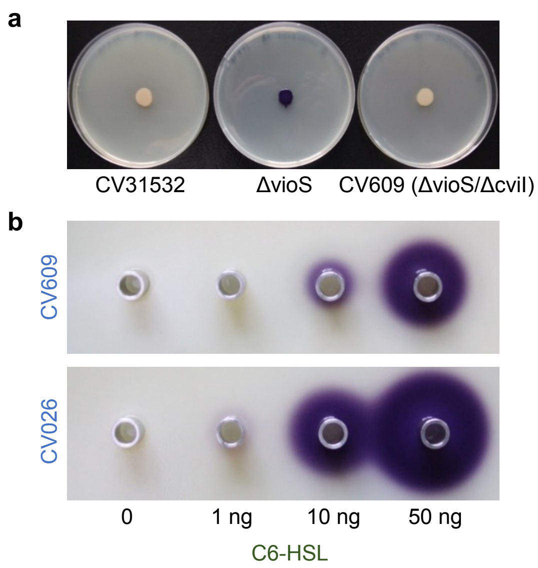

Supplementary Fig. 13. Construction and evaluation of CV609. **a** *vioS* and *cviI* in CV31532 were disrupted through homologous recombination to obtain CV609 (ΔvioS/ΔcviI), an AHL indicator strain with reduced sensitivity. **b** Sensitivity comparison of CV609 with CV026 to C6-HSL. Oxford cup assay was applied to detect violacein production induced by different amounts of C6-HSL dissolved in 70 μL of methanol. Data shown above are representative of two independent experiments that displayed similar results.

Supplementary Table 1. HPLC Conditions for the analysis and isolation of TOXAs

**Time (min)**	0	50	51	55	56	60
**ACN (%)**	37	37	100	100	37	37

Supplementary references

1. Liu, X. et al. A widespread response of Gram-negative bacterial acyl-homoserine lactone receptors to Gram-positive *Streptomyces* gamma-butyrolactone signaling molecules. *Sci. China Life Sci.* **64**, 1575-1589 (2021).
2. Zhao, C. et al. Oxazolomycin biosynthesis in *Streptomyces albus* JA3453 featuring an "acyltransferase-less" type I polyketide synthase that incorporates two distinct extender units. *J. Biol. Chem.* **285**, 20097-20108 (2010).
