# Supplementary material for: A visualization reporter system for characterizing antibiotic biosynthetic gene clusters expression with high-sensitivity
Source: Commun Biol. 2022 Sep 2;5:901. doi: 10.1038/s42003-022-03832-9 (PMC9440138; doi:10.1038/s42003-022-03832-9)
Supplement: Supplementary file 3 — Description of Additional Supplementary Files [file 42003_2022_3832_MOESM3_ESM.pdf]

Description of Additional Supplementary Files

File name: Supplementary Data 1
Description: Proteins encoded by *oxa* gene cluster.

File name: Supplementary Data 2
Description: Strains co-cultivated with 4.1101DRoxaG.

File name: Supplementary Data 3
Description: ^{1}H and ^{13}C NMR data of TOXA1.

File name: Supplementary Data 4
Description: ^{1}H and ^{13}C NMR data of TOXA5.

File name: Supplementary Data 5
Description: ^{1}H and ^{13}C NMR data of TOXA7.

File name: Supplementary Data 6
Description: The source data of Fig. 5d, f in the paper.

File name: Supplementary Data 7
Description: Bacterial strains used in this study.

File name: Supplementary Data 8
Description: Plasmids used in this study.

File name: Supplementary Data 9
Description: Primers used in this study.

File name: Supplementary Data 10
Description: The source data of Supplementary Fig. 2.

File name: Supplementary Data 11
Description: The source data of Supplementary Fig. 3b.

File name: Supplementary Data 12
Description: The source data of Supplementary Fig. 6b.
