# Supplementary material for: A visualization reporter system for characterizing antibiotic biosynthetic gene clusters expression with high-sensitivity
Source: Commun Biol. 2022 Sep 2;5:901. doi: 10.1038/s42003-022-03832-9 (PMC9440138; doi:10.1038/s42003-022-03832-9)
Supplement: Supplementary file 4 — Supplementary Data 1 [file 42003_2022_3832_MOESM4_ESM.pdf]

Supplementary Data 1. Proteins encoded by *oxa* gene cluster

**Protein**	**Size (aa)**	**Predicted function**	**Homolog in *ozm***	**Identity/ Positive (%/%)**
Orf-14	215	threonylcarbamoyl-AMP synthase		
Orf-13	227	protein-tyrosine-phosphatase		
Orf-12	462	serine hydroxymethyltransferase		
Orf-11	446	undecaprenyl/decaprenyl-phosphate alpha-*N*-acetylglucosaminyl 1-phosphate transferase		
Orf-10	132	Unknown		
Orf-9	269	F0F1 ATP synthase subunit A		
Orf-8	89	ATP synthase F0 subunit C		
Orf-7	188	F0F1 ATP synthase subunit B		
Orf-6	271	F0F1 ATP synthase subunit delta		
Orf-5	530	F0F1 ATP synthase subunit alpha		
Orf-4	305	F0F1 ATP synthase subunit gamma		
Orf-3	457	F0F1 ATP synthase subunit beta		
Orf-2	127	F0F1 ATP synthase subunit epsilon		
Orf-1	147	Unknown		
OxaB	379	glyceroyl transferase/phosphatase	OzmB	75/82
OxaC	321	3-oxoacyl-ACP synthase	OzmC	86/92
OxaD	366	acyl-CoA/acyl-ACP dehydrogenase	OzmD	85/89
OxaE	93	acyl carrier protein	OzmE	72/84
OxaF	226	class I SAM-dependent methyltransferase	OzmF	84/90
OxaG	285	3-hydroxyacyl-CoA dehydrogenase	OzmG	77/83
OxaH	7777	Hybrid NRPS/PKS	OzmH	74/80

OxaJ	2930	PKS	OzmJ	79/85
OxaK	1203	PKS	OzmK	79/85
OxaL	1979	NRPS	OzmL	54/65
OxaM	1096	Acyltransferase/oxidoreductase	OzmM	80/87
OxaN	5045	PKS	OzmN	71/76
OxaO	1192	NRPS	OzmO	72/78
OxaP	382	Unknown	OzmP	86/94
OxaQ	853	PKS	OzmQ	72/79
Orf1	229	LuxR family two component transcriptional regulator		
Orf2	401	two-component sensor histidine kinase		
Orf3	194	Unknown		
Orf4	190	cob(I)yrinic acid a,c-diamide adenosyltransferase		
Orf5	263	ABC transporter permease		
Orf6	247	ABC transporter ATP-binding protein		
Orf7	213	TetR/AcrR family transcriptional regulator		
Orf8	282	3-hydroxybutyryl-CoA dehydrogenase	OzmG	34/57
Orf9	107	anti-sigma factor antagonist		
Orf10	876	AAA family ATPase		
Orf11	220	Endonuclease NucS		
Orf12	130	Unknown		
Orf13	349	LLM class flavin-dependent oxidoreductase		
Orf14	107	Unknown		
Orf15	423	ABC transporter permease		
Orf16	440	ABC transporter ATP-binding protein		
