# Supplementary material for: A visualization reporter system for characterizing antibiotic biosynthetic gene clusters expression with high-sensitivity
Source: Commun Biol. 2022 Sep 2;5:901. doi: 10.1038/s42003-022-03832-9 (PMC9440138; doi:10.1038/s42003-022-03832-9)
Supplement: Supplementary file 5 — Supplementary Data 2 [file 42003_2022_3832_MOESM5_ESM.pdf]

Supplementary Data 2. Strains co-cultivated with 4.1101DRoxaG

Co-cultivation combination (CC)	Strains that were co-cultivated with 4.1101DRoxaG	Sources/references
CC1	*Streptomyces coelicolor* M1146	Ref.[1]
CC2	*Streptomyces albus* CGMCC 4.5716	CGMCC
CC3	*Streptomyces virginiae* ATCC 13161	ATCC
CC4	*Streptomyces griseus* IFO 13350	Ref.[2]
CC5	*Streptomyces venezuelae* ISP 5230	Ref.[3]
CC6	*Streptomyces fradiae* FXJ 1.408	Laboratory stock
CC7	*Streptomyces olivaceus* FXJ 8.021	Laboratory stock
CC8	*Streptomyces* sp. FXJ 1.088	Laboratory stock
CC9	*Streptomyces lividans* TK23	Ref.[4]
CC10	*Streptomyces ansochromogenes* 7100	Ref.[5]
CC11	*Streptomyces luridus* CGMCC 4.1115	CGMCC
CC12	*Bacillus subtilis* CGMCC 1.1630	CGMCC
CC13	*Staphylococcus aureus* CGMCC 1.89	CGMCC
CC14	*Bacillus cereus* CGMCC1.1626	CGMCC
CC15	*Staphylococcus epidermidis* ATCC 35984	Ref.[6]
CC16	*Streptococcus pneumoniae* 010	Ref.[6]
CC17	*Streptococcus pyogenes* #2	Ref.[6]
CC18	*Escherichia coli* JM109	Invitrogen
CC19	*Corynebacterium marinum* CGMCC 1.6998	CGMCC
CC20	*Corynebacterium glutamicum* CGMCC 1.299	CGMCC
CC21	*Rhodobacter blasticus* CGMCC 1.3365	CGMCC
CC22	*Rhodococcus coprophilus* CGMCC 4.1813	CGMCC
CC23	*Chaetomium* SP. TAN01	Laboratory stock

CC24	*Eupenicillium* SP. TAN02	Laboratory stock
CC25	*Aspergillus* SP. TAN03	Laboratory stock
CC26	*Aspergillus* SP. TAN04	Laboratory stock
CC27	*Aspergillus* SP. TAN05	Laboratory stock
CC28	*Penicillium* SP. TAN06	Laboratory stock
CC29	*Cladosporium* SP. TAN07	Laboratory stock
CC30	*Penicillium* SP. TAN08	Laboratory stock
CC31	*Coprinellus* SP. TAN09	Laboratory stock
CC32	*Phoma* SP. TAN10	Laboratory stock
CC33	*Aspergillus* SP. TAN11	Laboratory stock

Supplementary references

1. Gomez-Escribano, J.P. & Bibb, M.J. Engineering *Streptomyces coelicolor* for heterologous expression of secondary metabolite gene clusters. *Microb. Biotechnol.* **4**, 207-215 (2011).

2. Kato, J.Y., Miyahisa, I., Mashiko, M., Ohnishi, Y. & Horinouchi, S. A single target is sufficient to account for the biological effects of the A-factor receptor protein of *Streptomyces griseus*. *J. Bacteriol.* **186**, 2206-2211 (2004).

3. Yang, K., Han, L. & Vining, L.C. Regulation of jadomycin B production in *Streptomyces venezuelae* ISP5230: involvement of a repressor gene, *jadR2*. *J. Bacteriol.* **177**, 6111-6117 (1995).

4. Kieser, T., Bibb, M.J., Buttner, M.J., Chater, K.F. & Hopwood, D.A. *Practical Streptomyces Genetics* (John Innes Foundation Norwich, 2000).

5. Wang, W. et al. Identification of a butenolide signaling system that regulates nikkomycin biosynthesis in *Streptomyces*. *J. Biol. Chem.* **293**, 20029-20040 (2018).

6. Lu, C., Liao, G., Zhang, J. & Tan, H. Identification of novel tylosin analogues generated by a *wblA* disruption mutant of *Streptomyces ansochromogenes*. *Microb. Cell Fact.* **14**, 173 (2015).
