# Supplementary material for: A visualization reporter system for characterizing antibiotic biosynthetic gene clusters expression with high-sensitivity
Source: Commun Biol. 2022 Sep 2;5:901. doi: 10.1038/s42003-022-03832-9 (PMC9440138; doi:10.1038/s42003-022-03832-9)
Supplement: Supplementary file 6 — Supplementary Data 3 [file 42003_2022_3832_MOESM6_ESM.pdf]

Supplementary Data 3. ^{1}H and ^{13}C NMR data of TOXA1

**Position**	**δ ^{13}C**	**δ ^{1}H (mult., *J*)**
1	174.74	
2	43.77	2.37 (q, *J* = 7.3 Hz, 1H)
3	81.3	
4	83.15	3.33 (dd, *J* = 8.8, 4.3 Hz, 2H)
5	32.38	1.14 (dd, *J* = 8.6, 4.1 Hz, 1H), 2.04 – 1.85 (m, 1H)
6	37.22	1.59 (dt, *J* = 12.8, 6.3 Hz, 1H)
7	75.69	3.83 (dd, *J* = 10.2, 5.9 Hz, 1H)
8	134.98	5.67 – 5.57 (m, 2H)
9	130.38	6.17 – 6.10 (m, 2H)
10	130.73	6.17 – 6.10 (m, 2H)
11	130.31	5.67 – 5.57 (m, 2H)
12	40.9	3.73 (dd, *J* = 9.3, 5.1 Hz, 2H)
13	10.21	1.04 (d, *J* = 7.3 Hz, 3H)
14	16.57	0.87 (d, *J* = 6.7 Hz, 3H)
15	84.97	
16	81.81	4.80 (dd, *J* = 9.1, 2.8 Hz, 1H)
17	170.79	
1'	176.56	
2'	46.32	
3'	73.56	4.64 (d, *J* = 4.7 Hz, 1H)
4'	140.41	
5'	123.94	6.41 (d, *J* = 12.0 Hz, 1H)
6'	124.91	6.32 (t, *J* = 11.4 Hz, 1H)
7'	127.6	5.94 (t, *J* = 11.0 Hz, 1H)
8'	128.53	6.76 (dd, *J* = 14.5, 12.0 Hz, 1H)

9'	129.48	5.85 – 5.73 (m, 1H)
10'	28.73	3.56 (d, *J* = 6.9 Hz, 2H)
11'	151.03	
12'	122.49	6.91 (s, 1H)
13'	151.82	8.24 (d, *J* = 6.6 Hz, 1H)
14'	25.19	1.11 (s, 3H)
15'	22	0.98 (s, 3H)
16'	20.42	1.74 (s, 3H), 1.72 – 1.65 (m, 1H)
16-CH2	61	4.18 (ddd, *J* = 12.9, 9.1, 5.6 Hz, 1H),
		4.01 (ddd, *J* = 12.8, 5.6, 2.7 Hz, 1H)
16-CH2-OH		4.94 (t, *J* = 5.6 Hz, 1H)
NCH3	26.48	2.82 (s, 3H)
OCH3	56.39	3.17 (d, *J* = 5.5 Hz, 3H)
3-OH		5.53 (s, 1H)
7-OH		4.85 (d, *J* = 4.0 Hz, 1H)
3'-OH		5.48 (d, *J* = 4.7 Hz, 1H)
NH		7.67 (t, *J* = 5.6 Hz, 1H)
