# Supplementary material for: A visualization reporter system for characterizing antibiotic biosynthetic gene clusters expression with high-sensitivity
Source: Commun Biol. 2022 Sep 2;5:901. doi: 10.1038/s42003-022-03832-9 (PMC9440138; doi:10.1038/s42003-022-03832-9)
Supplement: Supplementary file 7 — Supplementary Data 4 [file 42003_2022_3832_MOESM7_ESM.pdf]

Supplementary Data 4. ^{1}H and ^{13}C NMR data of TOXA5

**Position**	**δ ^{13}C**	**δ ^{1}H (mult., *J*)**
1	174.92	
2	44.07	2.38 (q, *J* = 7.2 Hz, 1H)
3	81.17	
4	83.11	3.37 (t, *J* = 4.7 Hz, 1H)
5	32.45	1.16 (ddd, *J* = 15.2, 8.4, 4.2 Hz, 1H),
		1.97 (dt, *J* = 15.2, 5.2 Hz, 1H)
6	37.2	1.62 (dt, *J* = 12.5, 6.1 Hz, 1H)
7	75.69	3.85 (dd, *J* = 10.2, 5.8 Hz, 1H)
8	134.95	5.67 – 5.58 (m, 2H)
9	130.32	6.19 – 6.10 (m, 2H)
10	130.74	6.19 – 6.10 (m, 2H)
11	130.36	5.67 – 5.58 (m, 2H)
12	40.9	3.73 (dd, *J* = 9.7, 5.1 Hz, 2H)
13	10.27	1.06 (d, *J* = 7.3 Hz, 3H)
14	16.55	0.88 (d, *J* = 6.7 Hz, 3H)
15	84.02	
16	77.8	5.01 (q, *J* = 6.5 Hz, 1H)
17	170.66	
1'	176.58	
2'	46.3	
3'	73.59	4.64 (d, *J* = 4.7 Hz, 1H)
4'	140.41	
5'	123.95	6.42 (d, *J* = 11.9 Hz, 1H)
6'	124.9	6.33 (t, *J* = 11.4 Hz, 1H)
7'	127.6	5.94 (t, *J* = 11.1 Hz, 1H)

8'	128.52	6.75 (dd, *J* = 14.5, 11.8 Hz, 1H)
9'	129.45	5.83 – 5.76 (m, 1H)
10'	28.73	3.56 (d, *J* = 6.9 Hz, 2H)
11'	151.03	
12'	122.48	6.90 (s, 1H)
13'	151.79	8.24 (s, 1H)
14'	25.2	1.12 (s, 3H)
15'	22	0.99 (s, 3H)
16'	20.41	1.75 (s, 3H)
16-CH3	17.34	1.72 (d, *J* = 6.5 Hz, 3H)
NCH3	26.45	2.80 (s, 3H)
OCH3	56.4	3.17 (s, 3H)
3-OH		5.36 (s, 1H)
7-OH		4.86 (d, *J* = 4.0 Hz, 1H)
3'-OH		5.49 (d, *J* = 4.7 Hz, 1H)
NH		7.67 (t, *J* = 5.6 Hz, 1H)
