# Supplementary material for: A visualization reporter system for characterizing antibiotic biosynthetic gene clusters expression with high-sensitivity
Source: Commun Biol. 2022 Sep 2;5:901. doi: 10.1038/s42003-022-03832-9 (PMC9440138; doi:10.1038/s42003-022-03832-9)
Supplement: Supplementary file 8 — Supplementary Data 5 [file 42003_2022_3832_MOESM8_ESM.pdf]

Supplementary Data 5. ^{1}H and ^{13}C NMR data of TOXA7

**Position**	**δ ^{13}C**	**δ ^{1}H (mult., *J*)**
1	174.91	
2	44.06	2.38 (q, *J* = 7.2 Hz, 1H)
3	81.17	
4	83.11	3.37
5	32.46	1.97 (dt, *J* = 15.2, 5.2 Hz, 1H), 1.20 – 1.14 (m, 1H)
6	37.19	1.62 (dt, *J* = 12.5, 6.1 Hz, 1H)
7	75.67	3.84 (s, 1H)
8	134.96	5.62 (dt, *J* = 10.4, 6.3 Hz, 2H)
9	130.3	6.19 – 6.10 (m, 3H)
10	130.74	6.19 – 6.10 (m, 3H)
11	130.35	5.62 (dt, *J* = 10.4, 6.3 Hz, 2H)
12	40.87	3.80 – 3.65 (m, 2H)
13	10.28	1.06 (d, *J* = 7.3 Hz, 3H)
14	16.55	0.88 (d, *J* = 6.7 Hz, 3H)
15	84.02	
16	77.79	5.01 (q, *J* = 6.5 Hz, 1H)
17	170.66	
1'	176.57	
2'	46.26	
3'	74.02	4.61 (d, *J* = 3.8 Hz, 1H)
4'	139.62	
5'	129.06	5.95 (d, *J* = 11.4 Hz, 1H)
6'	128.88	6.60 (dd, *J* = 14.3, 11.6 Hz, 1H)
7'	131.4	6.19 – 6.10 (m, 3H)
8'	133.46	6.24 (dd, *J* = 14.9, 10.8 Hz, 1H)

9'	128.04	5.81 – 5.71 (m, 1H)
10'	28.52	3.52 (d, *J* = 6.7 Hz, 2H)
11'	150.99	
12'	122.62	6.91 (s, 1H)
13'	151.85	8.24 (s, 1H)
14'	25.15	1.12 (s, 3H)
15'	22.08	0.98 (s, 3H)
16'	20.23	1.69(3H,s)
16-CH3	17.35	1.71 (d, 3H)
N-CH3	26.45	2.80 (s, 3H)
O-CH3	56.41	3.17 (d, *J* = 5.7 Hz, 3H)
3-OH		5.36 (s, 1H)
7-OH		4.85 (d, *J* = 2.9 Hz, 1H)
3'-OH		5.48 (d, *J* = 4.4 Hz, 1H)
NH		7.67 (t, *J* = 5.6 Hz, 1H)
