# Supplementary material for: A visualization reporter system for characterizing antibiotic biosynthetic gene clusters expression with high-sensitivity
Source: Commun Biol. 2022 Sep 2;5:901. doi: 10.1038/s42003-022-03832-9 (PMC9440138; doi:10.1038/s42003-022-03832-9)
Supplement: Supplementary file 10 — Supplementary Data 7 [file 42003_2022_3832_MOESM10_ESM.pdf]

Supplementary Data 7. Bacterial strains used in this study

Strains	Description	Sources/references
Streptomyces		
S. ansochromogenes	*S. ansochromogenes* 7100, wild-type	Ref.[1]
S. longshengensis	*S. longshengensis* CGMCC 4.1101, wild-type	CGMCC
S. virginiae	*S. virginiae* CGMCC 4.1530, wild-type	CGMCC
S. griseus	*S. griseus* IFO 13350, wild-type	Ref.[2]
S. venezuelae	*S. venezuelae* ISP 5230, wild-type	Ref.[3]
S. coelicolor A3(2)	*S. coelicolor* A3(2), wild-type	Ref.[4]
S. lividans	*S. lividans* TK23, *spc-1*, SLP2$^-$, SLP3$^-$	Ref.[4]
7100NC	*S. ansochromogenes* 7100 containing pIJ10500K	This work
7100OEcviI	*S. ansochromogenes* 7100 containing pPhrdB-cviI	This work
7100OEcviI-12472	*S. ansochromogenes* 7100 containing pPhrdB-cviI-12472	This work
7100ovm-cviI	*S. ansochromogenes* 7100 containing pPovmOI-cviI	This work
7100ovm-cviI-NC	7100ovm-cviI containing pKC1139	This work
7100ovm-cviI-OEzw	7100ovm-cviI containing pKC1139::P$_{hrdB}$ZW	This work
7100ovm-cviIΔsabA	*sabA* disruption mutant of 7100ovm-cviI	This work
7100ang-cviI	*S. ansochromogenes* 7100 containing pPang1-cviI	This work
M1146	*S. coelicolor* A3(2) derivative, *act*$^-$, *red*$^-$, *cpk*$^-$, *cda*$^-$, SCP1$^-$, SCP2$^-$	Ref.[5]

M1146ovm-cviI	M1146 containing pPovmOI-cviI	This work
M1146ovm-cviI-NC	M1146ovm-cviI containing pKC1139	This work
M1146ovm-cviI-OEzw	M1146ovm-cviI containing pKC1139::P_{hrdB}ZW	This work
4.1101DRoxaG	*S. longshengensis* CGMCC 4.1101 derivative strain in which partial coding region of *oxaG* was replaced with *cviI-kanR* fusion	This work
4.1101DRoxaH	*S. longshengensis* CGMCC 4.1101 derivative strain in which partial coding region of *oxaH* was replaced with *cviI-kanR* fusion	This work
4.1101OEcviI	*S. longshengensis* CGMCC 4.1101 containing pPhrdB-cviI	This work
C. violaceum		
CV31532	*C. violaceum* ATCC 31532, wild-type	Ref.[6]
CV026	The indicator strain of AHLs, Tn5 mutant of CV31532	Ref.[6]
ΔvioS	*vioS* disruption mutant of CV31532	This work
CV609	ΔvioS/ΔcviI, *cviI* disruption mutant of ΔvioS	This work
CV12472	*C. violaceum* ATCC 12472, wild-type	Ref.[7]
ΔcviI-12472	*cviI-12472* disruption mutant of CV12472	Ref.[8]
E. coli		
JM109	Host strain for plasmid construction and	Invitrogen

	reporter system use	
ET12567/pUZ8002	ET12567 with helper plasmid pUZ8002 for conjugation between *E. coli* and *Streptomyces*	Ref.[4]
S17-1 λpir	Host strain able to transfer suicide plasmids requiring the Pir protein by conjugation to recipient cells	Ref.[9]
EJ532-4	*E. coli* JM109 derivative indicator strain of AHLs based on AHL QS of CV31532	Ref.[8]
**Others**		
B. subtilis	*Bacillus subtilis* CGMCC 1.1630, strain used for co-cultivation and bioassays	CGMCC
S. aureus	*Staphylococcus aureus* CGMCC 1.89, strain used for co-cultivation and bioassays	CGMCC
B. cereus	*Bacillus cereus* CGMCC1.1626, strain used for co-cultivation and bioassays	CGMCC
P. aeruginosa	*Pseudomonas aeruginosa* PA14, strain used for bioassays	Laboratory stock
B. cepacia	*Burkholderia cepacia* CGMCC 1.1813, strain used for bioassays	CGMCC
X. campestris	*Xanthomonas campestris* Xcc 8004, strain used for bioassays	Ref.[10]

Supplementary references

1. Wang, W. et al. Identification of a butenolide signaling system that regulates nikkomycin biosynthesis in *Streptomyces*. *J. Biol. Chem.* **293**, 20029-20040 (2018).
2. Kato, J.Y., Miyahisa, I., Mashiko, M., Ohnishi, Y. & Horinouchi, S. A single target is sufficient to account for the biological effects of the A-factor receptor protein of *Streptomyces griseus*. *J. Bacteriol.* **186**, 2206-2211 (2004).
3. Yang, K., Han, L. & Vining, L.C. Regulation of jadomycin B production in *Streptomyces venezuelae* ISP5230: involvement of a repressor gene, *jadR2*. *J. Bacteriol.* **177**, 6111-6117 (1995).
4. Kieser, T., Bibb, M.J., Buttner, M.J., Chater, K.F. & Hopwood, D.A. *Practical Streptomyces Genetics* (John Innes Foundation Norwich, 2000).
5. Gomez-Escribano, J.P. & Bibb, M.J. Engineering *Streptomyces coelicolor* for heterologous expression of secondary metabolite gene clusters. *Microb. Biotechnol.* **4**, 207-215 (2011).
6. McClean, K.H. et al. Quorum sensing and *Chromobacterium violaceum*: exploitation of violacein production and inhibition for the detection of *N*-acylhomoserine lactones. *Microbiology* **143**, 3703-3711 (1997).
7. Morohoshi, T., Kato, M., Fukamachi, K., Kato, N. & Ikeda, T. *N*-acylhomoserine lactone regulates violacein production in *Chromobacterium violaceum* type strain ATCC 12472. *FEMS Microbiol. Lett.* **279**, 124-130 (2008).
8. Liu, X. et al. A widespread response of Gram-negative bacterial acyl-homoserine lactone receptors to Gram-positive *Streptomyces* gamma-butyrolactone signaling molecules. *Sci. China Life Sci.* **64**, 1575-1589

(2021).

9. Wei, Q. et al. Diguanylate cyclases and phosphodiesterases required for basal-level c-di-GMP in *Pseudomonas aeruginosa* as revealed by systematic phylogenetic and transcriptomic analyses. *Appl. Environ. Microb.* **85** (2019).

10. Tang, D.J. et al. A SAM-I riboswitch with the ability to sense and respond to uncharged initiator tRNA. *Nat. Commun.* **11**, 2794 (2020).
