# Supplementary material for: A visualization reporter system for characterizing antibiotic biosynthetic gene clusters expression with high-sensitivity
Source: Commun Biol. 2022 Sep 2;5:901. doi: 10.1038/s42003-022-03832-9 (PMC9440138; doi:10.1038/s42003-022-03832-9)
Supplement: Supplementary file 11 — Supplementary Data 8 [file 42003_2022_3832_MOESM11_ESM.pdf]

Supplementary Data 8. Plasmids used in this study

Plasmids	Description	Sources/references
pSET152	Integrative vector, containing φC31 integrase gene, Apr^R	Ref.[1]
pSPhrdB-cviI	pSET152 derivative with *cviI* driven by P_{hrdB}	This work
pIJ10500	Integrative vector, a derivative of pMS82 containing φBT1 integrase gene, Hyg^R	Ref.[2]
pIJ10500K	Integrative vector, a derivative of pIJ10500, Hyg^R, Km^R	This work
pPhrdB-cviI	pIJ10500K derivative with *cviI* driven by P_{hrdB}	This work
pPhrdB-cviI-12472	pIJ10500K derivative with *cviI-12472* driven by P_{hrdB}	This work
pPovmOI-cviI	pIJ10500K derivative with *cviI* driven by P_{ovmOI}	This work
pPang1-cviI	pIJ10500K derivative with *cviI* driven by P_{ang1}	This work
pKC1139	Multiple-copy, temperature-sensitive *E. coli–Streptomyces* shuttle vector	Ref.[3]
pKC1139::P_{hrdB}ZW	pKC1139 derivative with *ovmZ* and *ovmW* driven by P_{hrdB}	Ref.[4]
pKDRoxaG	pKC1139 derivative used for replacing partial coding region of *oxaG* with *cviI-kanR* fusion	This work
pKDRoxaH	pKC1139 derivative used for replacing partial coding region of *oxaH* with *cviI-kanR* fusion	This work

pKC1139AD	pKC1139 derivative used for disruption of *sabA*	Ref.[5]
pKnock-Km	Conjugative suicide vector, Km^R	Ref.[6]
pKKDvioS	A DNA fragment containing the left and right flanks of *vioS* was inserted into pKnocK-Km; Used for disruption of *vioS* in CV31532	This work
pKKDcviI	A DNA fragment containing the left and right flanks of *cviI* was inserted into pKnocK-Km; Used for disruption of *cviI* in ΔvioS	This work

Supplementary references

1. Kieser, T., Bibb, M.J., Buttner, M.J., Chater, K.F. & Hopwood, D.A. *Practical Streptomyces Genetics* (John Innes Foundation Norwich, 2000).
2. Pullan, S.T., Chandra, G., Bibb, M.J. & Merrick, M. Genome-wide analysis of the role of GlnR in *Streptomyces venezuelae* provides new insights into global nitrogen regulation in actinomycetes. *BMC Genomics* **12**, 175 (2011).
3. Bierman, M. et al. Plasmid cloning vectors for the conjugal transfer of DNA from *Escherichia coli* to *Streptomyces* spp. *Gene* **116**, 43-49 (1992).
4. Xu, J. et al. Activation and mechanism of a cryptic oviedomycin gene cluster via the disruption of a global regulatory gene, *adpA*, in *Streptomyces ansochromogenes*. *J. Biol. Chem.* **292**, 19708-19720 (2017).
5. Wang, W. et al. Identification of a butenolide signaling system that regulates nikkomycin biosynthesis in *Streptomyces*. *J. Biol. Chem.* **293**, 20029-20040 (2018).

6. Devescovi, G. et al. Negative regulation of violacein biosynthesis in *Chromobacterium violaceum*. *Front. Microbiol.* **8**, 349 (2017).
