# Supplementary material for: A visualization reporter system for characterizing antibiotic biosynthetic gene clusters expression with high-sensitivity
Source: Commun Biol. 2022 Sep 2;5:901. doi: 10.1038/s42003-022-03832-9 (PMC9440138; doi:10.1038/s42003-022-03832-9)
Supplement: Supplementary file 12 — Supplementary Data 9 [file 42003_2022_3832_MOESM12_ESM.pdf]

Supplementary Data 9. Primers used in this study

Primers	Sequence (5'–3')	Purpose
SPHRDBF	GCTCTAGACCGCCTTCCGCCGGAAC	pSPhrdB-cviI construction
PHRDBR	GAACAACCTCTCGGAACGTTGAA	
I532F	GTGAAAAAGTTCTACTCGTTGC	
SI532R	CGGGATCCTCAATGCGAATAATCGTACTCA	
KANRF	CCCAAGCTTAAGGGCCTCGTGATACGC	pIJ10500K construction
KANRR	GGGGTACCGGCTAATGCACCCAGTAAGG	
PHRDBF	GGAATTCCATATGCCGCCTTCCGCCGGAAC	pPhrdB-cviI construction
I532R	GGACTAGTTCAATGCGAATAATCGTACTCA	
I472F	ATGAAAGATTTTTTTCCGCTG	pPhrdB-cviI-12472 construction
I472R	GGACTAGTTCAATGCGAATAATCGTACTCA	
POVMF	GGAATTCCATATGTCCTGCCGGACTCCGCGC	pPovmOI-cviI construction
POVMR	CCGCGCCTCCTTTCCTGTTG	
PANGF	GGAATTCCATATGCTGGGCGAGCGGATGGAC	pPang1-cviI construction
PANGR	CAAGTCTCCGTAGCCAAAGCCATAG	
DROXAGLF	GACTCTAGAGGATCCGCGGCCGCGCGCGATCG CCCCACTTGCTGTAACTGC	pKDRoxaG construction
DROXAGLR	CTTTTTCACTCAAGCCTCCTAATTTTGGTTGGTG ATCTCCAACTGACCCTGC	
DROXAGRF	TTGACGAGTTCTTCTGAGCGGGACTCTGGGCCG	

	CGGCTTCTACACCTACC	
DROXAGRR	AAACAGCTATGACATGATTACGAATTCGATCTT GACCGTGGTGGCGATG	
RBSI532F	AACCAAAATTAGGAGGCTTGAGTG	
RBSI532R	TCAATGCGAATAATCGTACTCACG	
PKANRF	CAATGGCGTGAGTACGATTATTCGCATTGAAA GGGCCTCGTGATACGCCT	
PKANRR	CCCAGAGTCCCGCTCAGAAG	
DROXAHLF	GACTCTAGAGGATCCGCGGCCGCGCGATCA GTGCCTTGACCTTGAGCCC	pKDRoxaH construction
DROXAHLR	CTTTTTCACTCAAGCCTCCTAATTTTGGTTATTG CGCCACAGTTCGTCCG	
DROXAHRF	TTGACGAGTTCTTCTGAGCGGGACTCTGGGCTA CCCGCTCCTGACCCTCG	
DROXAHRR	AAACAGCTATGACATGATTACGAATTCGATAC GTGCAGTTCGGTGTTGGC	
DVIOSLF	CCGGGCTGCAGGAATTCGATGCAGCCATCTTA GCGAGC	pKKDvioS construction
DVIOSLR	TTGGTTGTGCAGCAGCCA	
DVIOSRF	GCTGGCTGCTGCACAACCAAGAGTCGCTAACG CTGCTTAC	
DVIOSRR	ACGGTATCGATAAGCTTGATGGTGGTGGGGCT GAACAT	
DCVIILF	CCGGGCTGCAGGAATTCGATCGTGGCCGGGTA GAAGAAGC	pKKDcviI construction
DCVIILR	GAACGCCAGCAGGTCGAGC	
DCVIIRF	GCTCGACCTGCTGGCGTTCGAGGTCATTCGTTC	

	GCTACGG
DCVIIRR	CGGTATCGATAAGCTTGATCATCTGCAAATCCC ACATCCC
