# Supplementary material for: A visualization reporter system for characterizing antibiotic biosynthetic gene clusters expression with high-sensitivity
Source: Commun Biol. 2022 Sep 2;5:901. doi: 10.1038/s42003-022-03832-9 (PMC9440138; doi:10.1038/s42003-022-03832-9)
Supplement: Supplementary file 16 — Reporting Summary [file 42003_2022_3832_MOESM16_ESM.pdf]

nature research

Corresponding author(s): Huarong Tan

Last updated by author(s): Aug 11, 2022

Reporting Summary

Nature Research wishes to improve the reproducibility of the work that we publish. This form provides structure for consistency and transparency in reporting. For further information on Nature Research policies, see our Editorial Policies and the Editorial Policy Checklist.

Statistics

For all statistical analyses, confirm that the following items are present in the figure legend, table legend, main text, or Methods section.

n/a	Confirmed	
☐	☒	The exact sample size (*n*) for each experimental group/condition, given as a discrete number and unit of measurement
☐	☒	A statement on whether measurements were taken from distinct samples or whether the same sample was measured repeatedly
☐	☒	The statistical test(s) used AND whether they are one- or two-sided<br>*Only common tests should be described solely by name; describe more complex techniques in the Methods section.*
☒	☐	A description of all covariates tested
☒	☐	A description of any assumptions or corrections, such as tests of normality and adjustment for multiple comparisons
☐	☒	A full description of the statistical parameters including central tendency (e.g. means) or other basic estimates (e.g. regression coefficient) AND variation (e.g. standard deviation) or associated estimates of uncertainty (e.g. confidence intervals)
☐	☒	For null hypothesis testing, the test statistic (e.g. *F*, *t*, *r*) with confidence intervals, effect sizes, degrees of freedom and *P* value noted<br>*Give P values as exact values whenever suitable.*
☒	☐	For Bayesian analysis, information on the choice of priors and Markov chain Monte Carlo settings
☒	☐	For hierarchical and complex designs, identification of the appropriate level for tests and full reporting of outcomes
☒	☐	Estimates of effect sizes (e.g. Cohen's *d*, Pearson's *r*), indicating how they were calculated

Our web collection on statistics for biologists contains articles on many of the points above.

Software and code

Policy information about availability of computer code

Data collection	Agilent ChemStation 32.1.0.0 was used for data collection in HPLC assays.<br>Agilent MassHunter Workstation Software Qualitative Analysis B.03.01 was used for data collection in LC-HRMS assays.<br>TopSpin 3.5 was used for data collection in NMR assays.<br>Gen5 1.10 was used for data collection in bioluminescence intensity detection and optical density measurement.
Data analysis	antiSMASH 6.0.0 was used for secondary metabolite biosynthetic gene clusters analyses.<br>Blast online (NCBI) and Blast+ 2.8.0 were used for sequence alignment.<br>GraphPad Prism 8.0.1 was used for all graphing and analyses unless otherwise stated.<br>ImageJ 1.53e was used for violacein zone area calculation.

For manuscripts utilizing custom algorithms or software that are central to the research but not yet described in published literature, software must be made available to editors and reviewers. We strongly encourage code deposition in a community repository (e.g. GitHub). See the Nature Research guidelines for submitting code & software for further information.

Data

Policy information about availability of data

All manuscripts must include a data availability statement. This statement should provide the following information, where applicable:

- Accession codes, unique identifiers, or web links for publicly available datasets
- A list of figures that have associated raw data
- A description of any restrictions on data availability

All data supporting this work are available within this article and the Supplementary Information. The sequence data of oxa gene cluster have been deposited in NCBI GenBank with the accession number OM807121, and sequences data about ovm and ang gene clusters can be found from the genomic DNA of S.

ansochromogenes 7100 which is available in China National Microbiology Data Center (NMDC) with accession number of NMDC60029072. Sequences of all newly generated plasmids have been deposited in NMDC with accession numbers of NMDCN00011OL–NMDCN00011OU. Source data of Fig. 5d, f and Supplementary Figs. 2, 3b and 6b are provided in Supplementary Data 6 and 10–12. Further information or materials related to the findings of this study are available from the corresponding authors upon reasonable request.

Field-specific reporting

Please select the one below that is the best fit for your research. If you are not sure, read the appropriate sections before making your selection.

☒ Life sciences ☐ Behavioural & social sciences ☐ Ecological, evolutionary & environmental sciences

For a reference copy of the document with all sections, see nature.com/documents/nr-reporting-summary-flat.pdf

Life sciences study design

All studies must disclose on these points even when the disclosure is negative.

Sample size	No sample size calculation was performed for any experiments in this study. They were based on widely used sizes of previous publications (Myronovskyi, M. et al., Appl. Environ. Microbiol. 77, 5370-5383 (2011); Xiang, S.H. et al., Metab. Eng. 11, 310-318 (2009); Seyedsayamdost, M.R., Proc. Natl Acad. Sci. USA 111, 7266-7271 (2014)) to ensure that they are sufficient enough to achieve significant results.
Data exclusions	No data were excluded from the analyses.
Replication	All attempts at replication were successful.
Randomization	Randomization is not relevant to this study.
Blinding	Blinding is not relevant to our study.

Reporting for specific materials, systems and methods

We require information from authors about some types of materials, experimental systems and methods used in many studies. Here, indicate whether each material, system or method listed is relevant to your study. If you are not sure if a list item applies to your research, read the appropriate section before selecting a response.

Materials & experimental systems

n/a	Involved in the study	
☒	☐	Antibodies
☒	☐	Eukaryotic cell lines
☒	☐	Palaeontology and archaeology
☒	☐	Animals and other organisms
☒	☐	Human research participants
☒	☐	Clinical data
☒	☐	Dual use research of concern

Methods

n/a	Involved in the study	
☒	☐	ChIP-seq
☒	☐	Flow cytometry
☒	☐	MRI-based neuroimaging
